# Afrotropical Ophioninae (Hymenoptera, Ichneumonidae): an update of Gauld and Mitchell’s revision, including two new species and an interactive matrix identification key

**DOI:** 10.3897/zookeys.456.8140

**Published:** 2014-11-21

**Authors:** Pascal Rousse, Simon van Noort

**Affiliations:** 1 Natural History Department, Iziko South African Museum, PO Box 61, Cape Town, 8000, South Africa; 2 Department of Botany and Zoology, Evolutionary Genomics Group, Stellenbosch University, Private Bag X1, Stellenbosch 7602, South Africa; 3 Department of Biological Sciences, University of Cape Town, Private Bag, Rondebosch, 7701, South Africa

**Keywords:** Africa, distribution records, host records, identification keys, Madagascar, parasitoid wasp, systematics, taxonomy

## Abstract

The revision of the Afrotropical Ophioninae is updated, based on the examination of about 800–900 individuals in the South African and European museum collections. A robust interactive matrix key was built to provide quick and reliable identifications. The key is available online at http://www.waspweb.org. Two new species are described: *Dicamptus maxipol* **sp. n.** and *Enicospilus gauldetmitchellorum* **sp. n.** Numerous new distribution and biological records are provided, and noticeable morphological intra-specific variations are detailed. *Enicospilus batus* Gauld & Mitchell, **syn. n.** is considered as a junior synonym of *Enicospilus luebberti* (Enderlein).

## Introduction

The subfamily Ophioninae is one of the two major subfamilies of Ichneumonidae that have been extensively revised in the Afrotropical region. The revision of the Ophioninae is mainly due to Gauld and Mitchell (1978) where they keyed and provided detailed descriptions for the nearly 200 species they treated in the collections of the major natural history museums housing African material. They could thus emphasise the high endemism of this fauna, 98% of these species are not reported from outside of Subsaharan Africa.

It is of note that very few amendments have been brought to their work since the revision was published, except three new species descriptions (Gauld 1982; Rousse and Villemant 2012) and some phylogenetic rearrangements (Gauld 1980; Gauld 1985; Quicke et al. 2005). Meanwhile, several expeditions led by the Iziko South African Museum in southern and tropical Africa produced a large amount of new ophionine material available for investigation. Here we provide new taxonomic, biological and distribution data extracted from examination of this material. In addition to updating Gauld and Mitchell’s revision, an improved key in a new matrix format is providedto simplify the identification of Ophioninae in the region.

## Material and methods

### Depositories

BMNH Natural History Museum, London, UK (Gavin Broad).

CASC California Academy of Science, San Fransisco, USA (Brian Fisher).

MHNR Muséum d’Histoire Naturelle de La Réunion, Saint Denis, France (Sonia Ribes).

MNHN Muséul National d’Histoire Naturelle, Paris, France (Claire Villemant).

MRAC Muséum Royal de l’Afrique Centrale, Tervueren, Belgium (Eliane de Coninck).

NMSA KwaZulu-Natal Museum, Pietermaritzburg, South Africa (Burgert Muller).

SAMC Iziko South African Museum, Cape Town, South Africa (Simon van Noort).

### Photographs

Specimens were point mounted on black, acid-free card for examination (using a Leica M205C stereomicroscope with LED light source), photography and long term preservation. Images were acquired using the Leica LAS 4.4 imaging system, which comprised a Leica® Z16 microscope with a Leica DFC450 Camera with 0.63× video objective attached. The imaging process, using an automated Z-stepper, was managed using the Leica Application Suite V 4.4 software installed on a desktop computer. Lighting was achieved using techniques summarized in Buffington et al. (2005), Kerr et al. (2008) and Buffington and Gates (2009). All images presented in this paper are available at http://www.waspweb.org.

### Terminology and abbreviations

The terminology follows Gauld and Mitchell (1978), but we preferred to use here the terms mesosoma and metasoma rather than alitrunk and gaster. Most morphological terms are also defined on HymaToL and HAO websites. The following morphometric abbreviations are used:

B: body length, from torulus base to apex of metasoma (mm).

F: fore wing length, from tegula base to wing apex (mm).

ML (malar line index): shortest distance between eye and mandible / basal mandibular width.

CT (clypeus transversality index): distance between outer edges of tentorial pits / median height of clypeus.

POL (post-ocellar line index): shortest distance between posterior ocelli / posterior ocellus longest diameter.

OOL (oculo-ocellar line index): shortest distance between eye and posterior ocellus / posterior ocellus longest diameter.

FI (frontal index of head, Gauld and Mitchell 1978): maximum diameter of anterior ocellus / distance between eyes through maximum diameter of anterior ocellus.

Fl1–2 (relative length of flagellomeres 1 and 2): length of flagellomere 1 (annellus excluded) / length of flagellomere 2.

Fl20 (elongation index of 20^th^ flagellomere): length / width of flagellomere 20.

**Figure 1.**
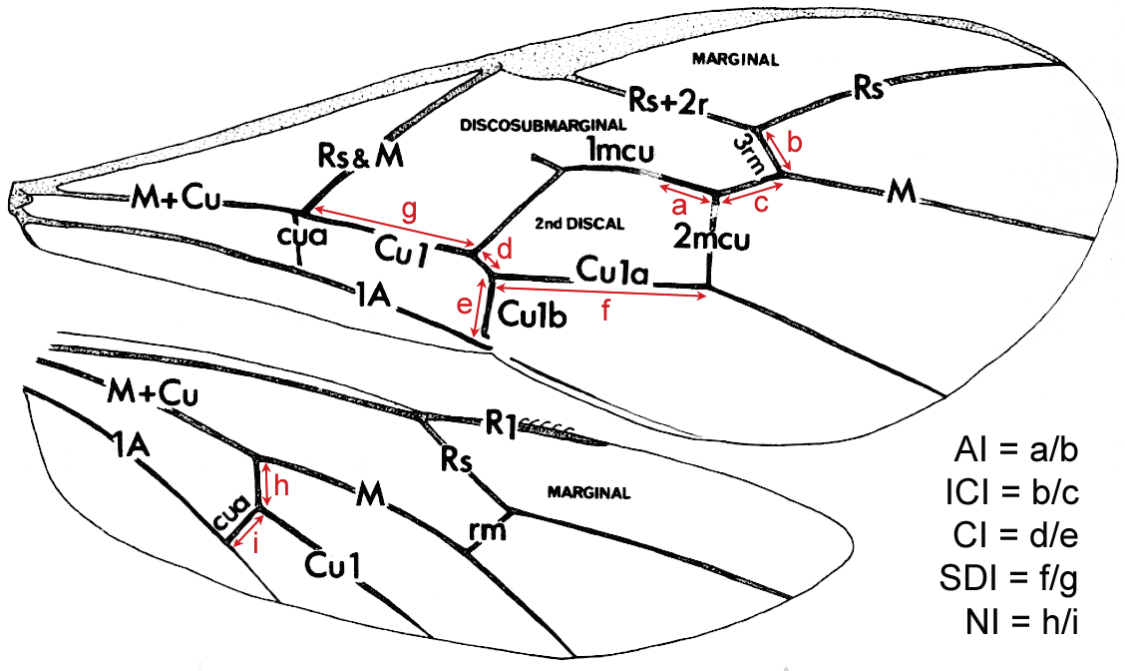
Wing venation terminology and alar indices (after Gauld and Mitchell 1978).

AI, CI, ICI, SDI, NI (alar indices, Gauld and Mitchell 1978): see Fig. 1.

### Material examined and key development

Nearly 500 individuals were examined in the SAMC collections. We examined 300–400 more housed in the BMNH, MRAC, and MNHN collections. An interactive matrix key was developed for their quick and reliable identification. This key was initially produced based on the data extracted from Gauld and Mitchell’s revision, and thereafter tested with the examined material. Uncertain identifications were cross-checked with Gauld and Mitchell’s key and descriptions, and the matrix key was then amended to fit the unreported variability. Each species was coded somewhat loosely to limit the risk of false negative results when selecting limital states of characters. Specific attention was paid to species described on a reduced number of individuals to deal with the subsequent reduced range of known variability.

## Results

Taking into account the taxonomic updates post Gauld and Mitchell’s revision, including the present one, we acknowledge here a total of 194 species of Ophioninae in the Afrotropical region oncluding *Skiapus coalescens* Morley 1917 which is now included in Ophioninae (Quicke et al. 2005). A few individuals could not be definitively attributed to a given species, but only two individuals are unambiguously new species, which are described below. We also provide a list of new distribution or biological records. Some of these are not actually new because they are mentioned in Gauld and Mitchell (1978), but all of them are not yet reported in the Taxapad reference database (Yu et al. 2012). Finally, we provide a list of significant morphological intra-specific variation, with one in particular leading us to consider *Enicospilus batus* Gauld & Mitchell, syn. n. as a junior synonym of *Enicospilus luebberti* (Enderlein).

### Identification keys

The matrix includes these 194 species and their known intra-specific variability. Furthermore, the dichotomous key provided in Gauld and Mitchell (1978) has been digitized and updated. Both keys are available at http://www.waspweb.org.

### Taxonomic descriptions

#### Dicamptus maxipol

Taxon classificationAnimaliaHymenopteraIchneumonidae

Rousse & van Noort sp. n.

http://zoobank.org/8C1C347B-4AD0-4FB2-A126-242C3FEA947C

Figs 2 –3

##### Type material

**(verbatim label data). HOLOTYPE** ♀: SOUTH AFRICA, W. Cape, West Coast Fossil Park, (5.5 km 270° W Langebaanweg) 32°57.759'S, 18°05.519'E, 9–16 Oct 2002, S. van Noort, Malaise trap LW02-R4-M96, Rehabilitated slimes dam, SAM-HYM-P049469 (SAMC).

##### Diagnosis.

Orange with inter-ocellar area, most of mesosoma and apex of metasoma black; mandible not twisted, with a central tuft of hairs; clypeus wide, long and flat in profile; antenna short and stout with 56 flagellomeres; mesosoma laterally coarsely punctate to rugose-punctate, dorsally densely and more finely punctate; mesoscutum with notaulus distinct and relatively long; mesopleuron with epicnemial carina not distinct above lower corner of pronotum; propodeum anteriorly densely punctate, posteriorly coarsely rugose-reticulate; disco-submarginal cell with fenestra developed but without distinct sclerite; fore tibia with dense and long spines on outer surface; fore tibial spur with a vestigial basal membrane.

##### Differential diagnosis.

Differentiated from all other *Dicamptus* species in the world by the absence of distinct sclerites in the disco-submarginal cell; in the Afrotropical region, it seems related to *Dicamptus neavei* Gauld & Mitchell, 1978, which shares the dense spines on the tibia, the exceptionally reduced ocelli and a somewhat similar colour pattern; *Dicamptus neavi* is, however, a tropical species with shorter antennae, a stouter metasoma, and distinctly different alar indices with a distinct proximal sclerite in the disco-submarginal cell. In Gauld and Mitchell’s key (1978), *Dicamptus maxipol* is included in the following modified first couplet:

**Table:** 

1	Fore wing with no alar scerite in the disco-submarginal cell; ocelli strongly reduced (FI < 0.25); South Africa	***Dicamptus maxipol***
–	Fore wing with one (rarely two) distinct sclerite(s) in the disco-submarginal cell; ocelli reduced to enlarged (FI ≥ 0.25)	**1a**
1a	Fore leg with 4th tarsal segment quadrate	

##### Description.

FEMALE (holotype). B 20.8; F 11.5; ML 1.2; CT 1.2; OOL 2.0; POL 1.2; FI 20%; F_1–2_ 1.7; F_20_ 1.2; AI 1.1; CI 0.5; ICI 0.7; SDI 1.1; NI 2.0.

*Color.* Orange interspersed with black; black: inter-ocellar area, entire mesosoma except for mesonotum and metanotum, base of tergite 1, tergite 5 and following, all coxae and trochanters except trochantelli; antenna orange, slightly darkening toward apex; wings hyaline, venation dark reddish to black except for pterostigma anteriorly light reddish.

*Head.* Mandible short and stout, without longitudinal groove, with a central tuft of long hairs, upper tooth barely longer than lower tooth; malar line long; clypeus long and wide, coarsely and densely punctate, rather flat in profile, somewhat swollen medially and ventrally, ventral margin strongly impressed; face strongly transverse, densely and coarsely punctate; frons rather smooth, upper head densely punctate; gena moderately swollen behind eyes; occipital carina complete and strong; antenna short and stout with 56 flagellomeres.

*Mesosoma.* Pronotum, mesopleuron and metapleuron coarsely and densely punctate, fading to rugose-punctate ventrally; anterior margin of pronotum simple; epicnemial carina short, indistinct above lower corner of pronotum; posterior transverse carina of mesosternum complete though ventrally weak; submetapleural carina not expanded anteriorly; mesoscutum densely and more finely punctate; notaulus long, moderate, distinct to anterior third of mesoscutum; scutellum densely punctate, carinate almost to apex; propodeum with anterior area densely punctate, anterior transverse carina complete, posterior area coarsely rugose-reticulate, abruptly declivous in profile and mid-posteriorly concave. *Wings.* Disco-submarginal cell with fenestra developed, without any distinct sclerite except a weak quadra centrally; *Rs+2r* hardly sinuate, slightly bent and thickened near pterostigma; *Rs&M* distal to *cu-a* by about its own width; hind wing with 7 hamuli. *Legs.* Fore tibia with dense and long spines on outer surface; fore tibial spur with a vestigial membrane basally to macrotrichial comb, membrane barely less than 0.1× length of spur; hind coxa in profile 1.8× as long as high; hind trochantellus mid-dorsally 0.2× as long as wide; hind tarsal claws symmetrical with 8 pectinae.

*Metasoma.* Slender; tergite 2 in profile 2.7× longer than high; thyridium large, oval, separated from anterior margin of tergite 2 by 1.3× its own length; ovipositor not reaching beyond metasomal apex.

MALE. Unknown.

**Figure 2.**
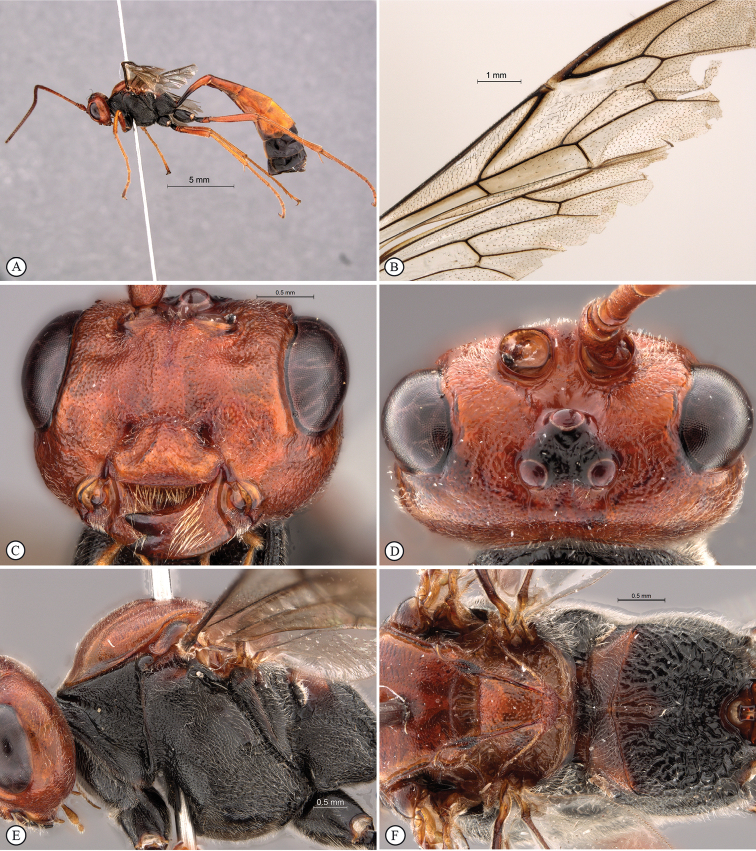
*Enicospilus maxipol* Holotype female. **A** habitus lateral view **B** wings **C** head anterio-ventral view **D** head dorsal view **E** head, mesosoma, lateral view **F** mesosoma, dorsal view.

**Figure 3.**
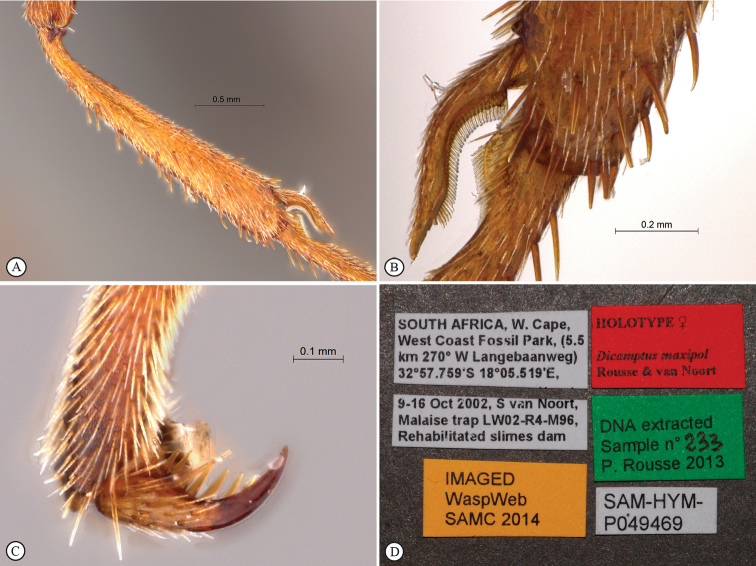
*Enicospilus maxipol* Holotype female. **A** fore tibia **B** fore tibial apex with spur, first tarsal segment **C** fore tarsal claws **D** data labels.

##### Etymology.

Named after the unusually reduced ocelli, and as a result the large POL. Noun in apposition.

##### Distribution.

South Africa (Western Cape).

#### Enicospilus gauldetmitchellorum

Taxon classificationAnimaliaHymenopteraIchneumonidae

Rousse & van Noort sp. n.

http://zoobank.org/5F861712-DBF0-41CC-9854-00DEB2913E86

Fig. 4

##### Type material

**(verbatim label data).** **HOLOTYPE** ♀: **Tanzania**, Mkomazi Game Reserve, Ibaya Camp, 3.58S 37.48E, 18 April 1996, light trap, S. van Noort, open *Combretum* bushland, SAM-HYM-P015183 (SAMC).

##### Diagnosis.

Yellow orange overall, head paler yellow; mandible with upper tooth distinctly longer than lower tooth; clypeus hardly convex in profile, its ventral margin barely concave and in-turned; occipital carinae complete; gena moderately swollen behind eye; ocelli moderately enlarged; antenna with 56 flagellomeres; pronotum unspecialized; mesopleuron and metapleuron closely and deeply punctate; epicnemial carina laterally indistinct; posterior transverse carina of mesosternum complete and noticeably strong; submetapleural carina slightly broadened anteriorly; notaulus vestigial; propodeum basally punctate, posteriorly coarsely and concentrically striate; fore wing without any sclerite in disco-submarginal cell; fore tibia with dense spines on outer surface; thyridium very shallow.

##### Differential diagnosis.

Readily differentiable from all other *Enicospilus* in Afrotropical, Oriental and Australasian regions by the combination of the absence of alar sclerites and the dense spines on fore tibia. The swollen genae and the wing venation make it somewhat related to *Enicospilus leucocotis*, but this latter is strongly larger, with only sparse spines on tibia and slenderer antenna. In Gauld and Mitchell’s key (1978), *Enicospilus gauldetmitchellorum* is included in the following modified eighth couplet:

**Table:** 

8	Fore tibia with dense and long spines, spines basally far closer than their own mean length; Tanzania	***Enicospilus gauldetmitchellorum***
–	Fore tibia with distinctly sparser spines, or no spine	**8a**
8a	Head, when viewed dorsally	

##### Description.

FEMALE (holotype). B 18.8; F 11.5; ML 0.3; CT 1.6; OOL 0.1; POL 0.4; FI 50%; F_1–2_ 1.4; F_20_ 2.2; AI 0.6; CI 0.7; ICI 0.6; SDI 1.3; NI 2.8.

*Color.* Yellowish orange overall with face and orbits paler yellow and apex of metasoma slightly infuscate.

*Head.* Mandible basally constricted, apically parallel-sided and slightly twisted, with upper tooth distinctly longer than lower tooth (greatly worn by abrasion in holotype); outer mandibular surface sparsely setose, without longitudinal groove; labrum 0.3× as long as wide; clypeus in profile hardly convex, its ventral margin barely concave and in-turned; clypeus and face finely and moderately densely punctate; gena moderately swollen behind eye; occipital carina complete; ocelli slightly enlarged; antenna with 56 flagellomeres.

*Mesosoma.* Pronotum mid-dorsally long, anterior margin simple; mesoscutum densely punctate, notaulus vestigial; scuto-scutellar groove smooth; scutellum densely and shallowly punctate, barely longer than basally wide, carinate to near its apex; mesopleuron and metapleuron closely and deeply punctate, punctures arranged longitudinally but without distinct striation; epicnemial carina short, indistinct above lower corner of pronotum; submetapleural carina weakly broadened anteriorly; posterior transverse carina of mesosternum complete and strong; propodeum with anterior area finely, shallowly and densely punctate, anterior transverse carina complete, posterior area coarsely and concentrically striate *Wings.* Disco-submarginal cell with fenestra developed, without any distinct sclerite; *Rs+2r* sinuate; *cu-a* basal to *Rs&M* by 0.3× *cu-a* length; hind wing with 6 hamuli and 1A basally straight. *Legs.* Fore tibia with numerous dense and long spines on outer surface, basally separated by far less than their own length; hind coxa elongate, in profile 2.4× as long as high; hind trochantellus mid-dorsally 0.2× as long as wide, its apical margin simple; hind tarsal claws symmetrical with 8 pectinae, pectinae long and acute.

*Metasoma.* Slender; tergite 2 in profile 3.2× longer than high; thyridium very shallow, elongate, separated from anterior margin of tergite 2 by 1.7× its own length; ovipositor acute not reaching beyond metasomal apex.

MALE. Unknown.

**Figure 4.**
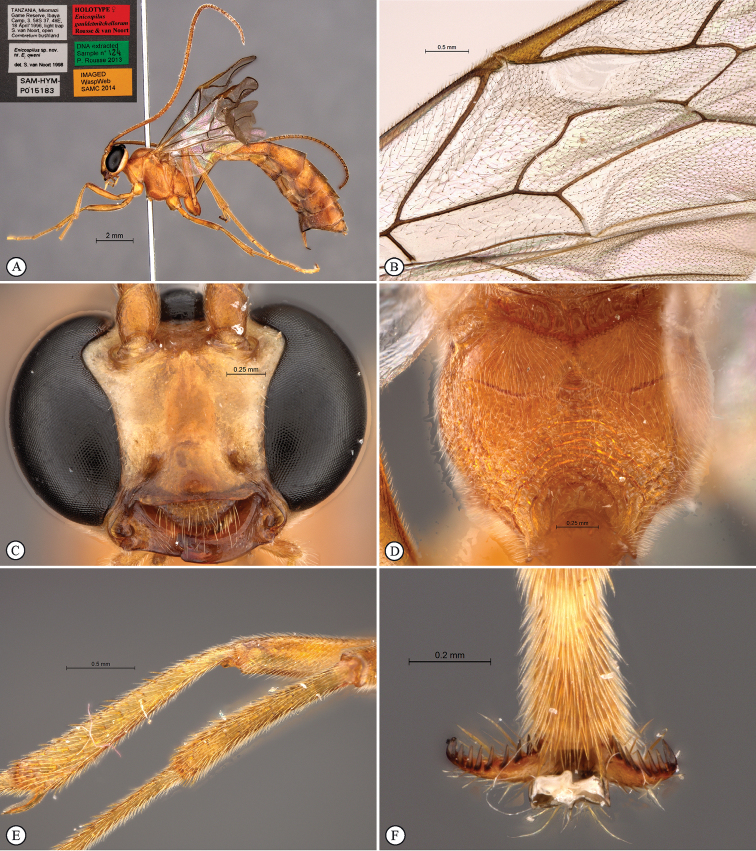
*Enicospilus gauldetmitchellorum* Holotype female. **A** habitus lateral view **B** wings **C** head anterio-ventral view **D** propodeum dorsal view **E** fore tibia, mid tibia **F** fore tarsal claws.

##### Etymology.

This species was probably mentioned in Gauld and Mitchell (1978) as an undescribed specimen close to *Enicospilus leucocotis*. Let give Gauld what belongs to Gauld (updated after Mark 12:17).

##### Distribution.

Tanzania.

### New distribution records

Provided are the verbatim label data. Only unambiguous identifications are listed. All geographical coordinates are also available on a separate file as Suppl. material 1. If not indicated on the labels, the coordinates were found on Fuzzy Gazeeter http://isodp.hof-university.de/fuzzyg/query/ and Google Earth http://www.google.com/earth/

*Dicamptus pulchellus* (Morley, 1912). **The Gambia:** 1♀ Kombo Nth district, Bilijo Forest Park, xi.1992, M. Söderlung coll., SAM-HYM-P049471 (SAMC).

*Euryophion latipennis* (Kirby, 1896). **South Africa:** 1♀ Kwazulu-Natal, Itala Game Reserve, xii.1992, S. van Noort coll., SAM-HYM-P044187 (SAMC); 1♀ 1♂ same label data except: xii.1999, SAM-HYM-P044163 and SAM-HYM-P044185 (SAMC); **Zambia:** 1 specimen [apex of metasoma lacking] Southern Province, Choma Nansa farm xii.1993, A.J. Gardiner coll., SAM-HYM-P044072 (SAMC).

*Laticoleus palpalis* Gauld & Mitchell, 1978. **Kenya:** 1♀ Eastern Province, Kenplains, x.1984, C.F. Dewhurst coll. (BMNH).

*Laticoleus unicolor* (Szépligeti, 1908). **Botswana:** 1♀ Xugana [verbatim label data, locality unknown], xi.1979, B.H. Lamoral coll., SAM-HYM-P049474 (SAMC).

*Lepiscelus distans* (Seyrig, 1935). **South Africa:** 1♀ Kwazulu-Natal, Itala Game Reserve, xii.1999, S. van Noort coll, SAM-HYM-P044186 (SAMC); 1♀ Limpopo, junction Crocodile and Marico Rivers, ii.1918, R. Tucker coll., SAM-HYM-P006194 (SAMC); 2♂♂ Mpumalanga, Nelspruit, i.1939, R.F. Lawrence coll., SAM-HYM-P006193 (SAMC); **Zimbabwe:** 1♂ Essexvale, ii.1963, SAM-HYM-P006228 (SAMC).

*Skiapus coalescens* (Morley, 1917). **The Gambia:** 1♀ Kombo Nth district, Bilijo Forest Park, xi.1992, M. Söderlung coll., SAM-HYM-P049477 (SAMC).

*Enicospilus albiger* (Kriechbaumer, 1894). **Zambia:** 1♂ South Luangwa near. Mfuwe, xii.2011, A. Gumovsky coll., SAM-HYM-P049484 (SAMC).

*Enicospilus babaulti* (Seyrig, 1935). **Malawi:** 1♀ Nyika National Park, Juniper forest, ix.1999, R.J. Murphy coll., SAM-HYM-P021341 (SAMC); **South Africa:** 1 specimen [metasoma lacking] ii.1917, C.J. Swierstra coll., SAM-HYM-P001398 (SAMC); **Zimbabwe:** 1♀ Chirinda forest, xi.1955, SAM-HYM-P006247 (SAMC).

*Enicospilus bebelus* Gauld & Mitchell, 1978. **Gabon:** 1♀ Province Ogoové–Maritime, Réserve des Monts Doudou, iii.2000, S. van Noort coll., SAM-HYM-P041707 (SAMC).

*Enicospilus betanimenus* (Saussure, 1892). **Ethiopia:** 2♀♀ Adola, xi.1941, SAM-HYM-P047374 and SAM-HYM-P006253 (SAMC); **Zimbabwe:** 2♀♀ Bulawayo ii.1971, D.K.B. Wheeler coll, SAM-HYM-P006286 (SAMC).

*Enicospilus bicoloratus* Cameron, 1912. **Zimbabwe:** 1♀ Matopos, ii.1963, SAM-HYM-P006265 (SAMC).

*Enicospilus camerunensis* (Enderlein, 1921). **Mayotte:** 1♀ Dembéni, iii.2013, G. Cazenove coll. (MHNR).

*Enicospilus divisus* (Seyrig, 1935). **Uganda:** 1♂ Kibale National Park, Kanyawara, viii.2008, S.van Noort coll., SAM-HYM-P049506 (SAMC).

*Enicospilus drakensbergi* Gauld & Mitchell, 1978. **Tanzania:** 1♂ South Pare Mountains, alt. c. 1700m, xi.1995, S. van Noort coll., SAM-HYM-P014698 (SAMC).

*Enicospilus equatus* Gauld & Mitchell, 1978. **Central African Republic:** 2♀♀ 1♂ Préfecture Sangha-Mbaéré, Réserve Spéciale de Forêt Dense de Dzanga-Sangha, v.2001, S. van Noort coll., SAM-HYM-P049510–P049512 (SAMC).

*Enicospilus finalis* Gauld & Mitchell, 1978. **Central African Republic:** 5♀♀ Préfecture Sangha-Mbaéré, Parc National de Dzanga-Ndoki, v.2001, S. van Noort coll., SAM-HYM-P049514 –P049517 (SAMC); **Mozambique:** 1 specimen [apex of metasoma broken] Mt Gorongoza, ix.1957, SAM-HYM-P006229 (SAMC).

*Enicospilus oculator* Seyrig, 1935. **Zimbabwe** 1♀ Tuli, v.1959, SAM-HYM-P006232 (SAMC).

*Enicospilus hova* Gauld & Mitchell, 1978. **Uganda:** 1♂ Kibale National Park, Kanyawara, viii.2008, S.van Noort coll., SAM-HYM-P049513 (SAMC).

*Enicospilus luebberti* (Enderlein, 1914). 4♀♀ **Botswana:** 1♀ Xugana [verbatim label data, locality unknown], xi.1979, B.H. Lamoral coll. (NMSA).

*Enicospilus mamatsus* Gauld & Mitchell, 1978. **South Africa:** 1♀ Northern Cape, Sterboom farm, 1599 m, v–vii 2010, S. van Noort, SAM-HYM-P054077 (SAMC).

*Enicospilus mnous* Gauld & Mitchell, 1978. **Tanzania:** 3♀♀, Mkomazi Game Reserve, xi.1995, H.G. Robertson coll. and S. van Noort colls, SAM-HYM-P014159, SAM-HYM-P014161 and SAM-HYM-P014170 (SAMC); 2♀♀ same label data except: iv.1996, S. van Noort coll., SAM-HYM-P014156 and SAM-HYM-P014706 (SAMC).

*Enicospilus nesius* Gauld & Mitchell, 1978. **Central African Republic:** 1♀ Préfecture Sangha-Mbaéré, Parc National de Dzanga-Ndoki, v.2001, S. van Noort coll., SAM-HYM-P054079 (SAMC).

*Enicospilus pallidus* (Taschenberg, 1875). **Tanzania:** 8♀♀, Mkomazi Game Reserve, xi–xii.1995 and iv.1996, S. van Noort coll., SAM-HYM-P014157–P0141578, SAM-HYM-P014171–P014175 and SAM-HYM-P015200 (SAMC).

*Enicospilus polemus* Gauld, 1982. **South Africa:** 1♀ Kwazulu-Natal, Itala Game Reserve, xii.1999, S. van Noort coll., SAM-HYM-P044207 (SAMC); **Tanzania:** 1♀ Mkomazi Game Reserve, iv–v.1996, S. van Noort coll., SAM-HYM-P015666 (SAMC).

*Enicospilus quietus* (Seyrig, 1935). **Namibia:** 1♀ Namib-Naukluft Park, x.1997, S. van Noort coll., SAM-HYM-P020721 (SAMC); 1♂ Otavi, xii.1918, R.M. Lightfoot coll., SAM-HYM-P006278 (SAMC); 1♀ Ondangua Ovamboland, 1921, K.H. Bernard coll., SAM-HYM-P006199 (SAMC); 2 specimens [metasomas lacking] Otjiperongo, i.1931, J.S. Brown coll., SAM-HYM-P047375 (SAMC).

*Enicospilus rubens* (Toquinet, 1896). **Madagascar:** 1♀ Majunga Province, Maintirano District, iii.2008, M.Irwin and R.Harin’Hala colls, MG-44-26 (CASC).

*Enicospilus rundiensis* Bischoff, 1915. **Namibia:** 1♀ 1♂ Kaross, 1925, SAM-HYM-P001381 (SAMC); 1♀ Warmbad, 1925, SAM-HYM-P001382 (SAMC); 1 specimen [apex of metasoma broken] Narubis, 1921, K.H. Barnard coll., SAM-HYM-P006277 (SAMC); 1 specimen [metasoma lacking] Otjiperongo, i.1931 J.S. Brown coll., SAM-HYM-P006276 (SAMC); **Zimbabwe:** 1♀ Harare, vi.1961, SAM-HYM-P006225 (SAMC).

*Enicospilus ruscus* Gauld & Mitchell, 1978. **Kenya:** 1♂ Nguruman, vii.2008, S. van Noort coll., SAM-HYM-P054106 (SAMC).

### New host records

*Enicospilus betanimenus* (Saussure, 1892). 2♀♀ from Zimbabwe SAM-HYM-P006286 (SAMC) ex *Achaea catella* Guenée, 1852 (Lepidoptera: Noctuidae).

*Enicospilus dubius* (Tosquinet, 1896). 2♀♀ from South Africa (SAMC SAM-HYM-P001508) ex *Ctenoplusia limbirina* (Guenée) (Lepidoptera: Noctuidae).

*Enicospilus dolosus* (Tosquinet, 1896). 1♀ from Reunion (M. Bippus, *com. pers.*) ex *Anomis flava* (Fabricius) (Lepidoptera: Noctuidae).

*Enicospilus leucocotis* (Tosquinet, 1896). 2♀♀ from South Africa (SAMC SAM-HYM-P046967 and SAM-HYM-P046968) ex *Mesocelis montana* (Hübner) (Lepidoptera: Lasiocampidae).

*Enicospilus mauritii* (Saussure, 1892). 1♂ from Reunion ex *Callopistria maillardi maillardi* (Guenée) (Lepidoptera: Noctuidae) feeding on *Dryopteris bernieri* (Pteridophyta: Dryopteridaceae) (Robert 2012).

*Enicospilus luebberti* (Enderlein, 1914). 1♂ from South Africa (SAMC SAM-HYM-P006196) ex *Graphania atavistis* (Hampson) (Lepidoptera: Noctuidae).

### Morphological variations

*Enicospilus bebelus* Gauld & Mitchell, 1978. 1♂ from Central African Republic (SAMC SAM-HYM-P049492) and 1♀ from Gabon (SAMC SAM-HYM-P041707) with mesosoma interspersed with dark testaceous and black markings, and tergite 1 basally black; otherwise similar to original description.

*Enicospilus oculator* Seyrig, 1935. 1♀ from Zimbabwe (SAMC SAM-HYM-P006232) with central sclerite totally absent, upper tooth twice as long as lower tooth, and numeric indices slightly different: FI 80%, AI 1.0, fore wing length 14 mm. Otherwise similar to Gauld and Mitchell (1978) description.

*Enicospilus grandiflavus* Townes, 1973. 1♀ from South Africa (SAMC SAM-HYM-P049521) with entire head strongly darkened, nearly black. Otherwise similar to Gauld and Mitchell (1978) description.

*Enicospilus expeditus* (Tosquinet, 1896). 1♀ 1♂ from South Africa (SAMC SAM-HYM-P054068) with hind tarsal claws less pectinate than figured in Gauld and Mitchell (1978), and with tergite 1 basally black and mesosoma largely interspersed with dark markings. Otherwise similar to Gauld and Mitchell (1978) description.

*Enicospilus luebberti* (Enderlein, 1914). Numerous specimens from South Africa (SAMC-HYM-P006210–21, P001454–55, P006196 and P054076), Bostwana (NMSA), and Kenya (BMNH) showed the following non-correlated variations: inter-ocellar area and metasomal apex yellowish-orange to totally black, antenna with 48–62 flagellomeres, longitudinal groove on mandible more or less impressed, proximal sclerite more or less elongate and central sclerite variously sclerotized. These variations encompass the definition of *Enicospilus batus* Gauld & Mitchell, 1978, syn. n., described on a single specimen, which is hereby recognized as a junior synonym of *Enicospilus luebberti*.

## Supplementary Material

XML Treatment for Dicamptus maxipol

XML Treatment for Enicospilus gauldetmitchellorum

## Supplementary materials

Supplementary material 1 Afrotropical Ophioninae: coordinates of the new distribution records. Data type: distribution data Explanation note: Summary of the collection localities and their geographical data listed as new distribution records for Afrotropical Ophioninae Copyright notice: This dataset is made available under the Open Database License (http://opendatacommons.org/licenses/odbl/1.0/). The Open Database License (ODbL) is a license agreement intended to allow users to freely share, modify, and use this Dataset while maintaining this same freedom for others, provided that the original source and author(s) are credited.Pascal Rousse, Simon van Noort

## References

[B1] Buffington ML Burks R McNeil L (2005) Advanced techniques for imaging microhymenoptera. American Entomologist 51: 50–54.

[B2] Buffington ML Gates M (2009) Advanced imaging techniques II: using a compound microscope for photographing point–mount specimens. American Entomologist 54: 222–224.

[B3] Cameron P (1912) On the Hymenoptera from Belgian Congo in the Congo Museum, Tervueren. Annales de la Société Entomologique de Belgique 56: 357–401.

[B4] Enderlein G (1914) Hymenoptera IV: Ichneumonidae. In: Michaelsen W (Ed.) Beiträge Zur Kenntnis Der Land-Und Süsswasserfauna Deutsch-Südwestafrikas. Band 1 Hamburg, Germany, 11–233. Biodiversity Heritage Library.

[B5] Enderlein G (1921) Beiträge zur Kenntnis aussereuropäischer Ichneumoniden V. Über die Familie Ophionidae. Stettiner Entomologische Zeitung 82: 3–45.

[B6] Gauld ID (1980) An analysis of the classification of the *Ophion* genus-group (Ichneumonidae). Systematic Entomology 5: 59–82. doi: 10.1111/j.1365-3113.1980.tb00400.x

[B7] Gauld ID (1982) A revised key of the *Enicospilus antefurcalis* (Szépligeti) (Hymenoptera: Ichneumonidae) species group of the Afrotropical region. Bulletin of Entomological Research 72: 33–38. doi: 10.1017/S0007485300050264

[B8] Gauld ID (1985) The phylogeny, classification and evolution of parasitic wasps of the subfamily Ophioninae (Ichneumonidae). Bulletin of the British Museum (Natural History), Entomology series 51: 61–185.

[B9] Gauld ID Mitchell PA (1978) The taxonomy, distribution and host preferences of African parasitic wasps of the subfamily Ophioninae (Hymenoptera, Ichneumonidae). Commonwealth Institute of Entomology, Slough, England. Waspweb.

[B10] Kerr PH Fisher EM Buffington ML (2008) Dome lighting for insect imaging under a microscope. American Entomologist 54: 198–200.

[B11] Kirby WF (1896) A list of the Orthoptera, Hymenoptera and Hemiptera collected by Miss Kingley on the River Ogove, with descriptions of some new genera and species. Ann. Mag. Nat. Hist. 18: 257–269. doi: 10.1080/00222939608680451

[B12] Kriechbaumer J (1894) Hymenoptera Ichneumonidae a medico nautico Dr. Joh. Brauns in itinere secundo ad oras Africae lecta. Berliner Entomologische Zeitschrift 39: 297–318. doi: 10.1002/mmnd.18940390215

[B13] Morley C (1912) A revision of the Ichneumonidae based on the collection in the British Museum (Natural History) with descriptions of new genera and species Part I. Tribes Ophionides and Metopiides. British Museum, London, 88 pp.

[B14] Morley C (1917) On some South African Ichneumonidae in the collection of the South African Museum. Annals of the South African Museum 17: 191–229.

[B15] Quicke DLJ Fitton MG Broad G Crocker B Laurenne NM Miah MI (2005) The parasitic wasp genera *Skiapus*, *Hellwigia*, *Nonnus*, *Chriodes*, and *Klutiana* (Hymenoptera, Ichneumonidae): recognition of the Nesomesochorinae stat. rev. and Nonninae stat. n. and transfer of *Skiapus* and *Hellwigia* to the Ophioninae. Journal of Natural History 39: 2559–2578. doi: 10.1080/00222930500102546

[B16] Robert Y (2012) La fougère, le papillon et la guèpe (Pteridophyta: Dryopteridaceae: Lepidoptera: Noctuidae; Hymenoptera: Ichneumonidae, Ophioninae). Cahiers Scientifiques de l’Océan Indien Occidental 3: 19–20.

[B17] Rousse P Villemant C (2012) Ichneumons in Reunion Island: a catalogue of the local Ichneumonidae (Hymenoptera) species, including 15 new taxa and a key to species. Zootaxa 3278: 1–57. 10.11646/zootaxa.3616.6.1 24758826

[B18] Saussure Hd (1892) Histoire naturelle des Hymenoptères. Histoire Physique Naturelle et Politique de Madagascar 20: 1–590.

[B19] Seyrig A (1935) Hymenoptera. II. Ichneumonidae: Cryptinae, Pimplinae, Tryphoninae et Ophioninae. Mission Scientifique de l’Omo 3: 1–103.

[B20] Szépligeti G (1908) Hymenoptera: Braconidae & Ichneumonidae. Wissenschaftliche Ergebnisse der Schwedischen Zoologischen Expedition nach dem Kilimandjaro, dem Meru und den umgebenden Massaisteppen 16: 25–96.

[B21] Taschenberg EL (1875) Zur Kenntnis der Gattung *Ophion* Fab. Zeitung Für Gesammten Naturwissenschaften 46: 421–438.

[B22] Tosquinet J (1896) Contributions à la faune entomologique de l’Afrique. Ichneumonides. Mémoires de la Société Entomologique de Belgique 5: 1–430.

[B23] Townes HK Townes M (1973) A catalogue and reclassification of the Ethiopian Ichneumonidae. Memoirs of the American Entomological Institute 19: 1–416.

[B24] Yu DSK van Achterberg C Horstmann K (2012) Taxapad 2012. Ichneumonoidea 2011. Database on flash-drive, Ottawa, Canada http://www.taxapad.com

